# Effect of Oxygen and Redox Potential on Glucose Fermentation in *Thermotoga maritima* under Controlled Physicochemical Conditions

**DOI:** 10.1155/2010/896510

**Published:** 2011-02-24

**Authors:** Raja Lakhal, Richard Auria, Sylvain Davidson, Bernard Ollivier, Alain Dolla, Moktar Hamdi, Yannick Combet-Blanc

**Affiliations:** ^1^UMR D180, IRD, ESIL, Universités de Provence et de la Méditerranée, 163 Avenue de Luminy, Case 925, 13288 Marseille Cedex 09, France; ^2^Unité Interactions et Modulateurs de Réponses, IFR88, CNRS, 31 Chemin Joseph Aiguier, 13402 Marseille Cedex 20, France; ^3^Laboratoire d'Ecologie et de Technologie Microbienne, Institut National des Sciences Appliquées et de Technologies (INSAT), Université 7 Novembre Carthage, 2 Boulevard de la Terre, BP 676, 1080 Tunis, Tunisia

## Abstract

Batch cultures of *Thermotoga maritima* were performed in a bioreactor equipped with instruments adapted for experiments performed at 80°C to mimic the fluctuating oxidative conditions in the hot ecosystems it inhabits. When grown anaerobically on glucose, *T. maritima* was shown to significantly decrease the redox potential (Eh) of the culture medium down to about −480 mV, as long as glucose was available. Addition of oxygen into *T. maritima* cultures during the stationary growth phase led to a drastic reduction in glucose consumption rate. However, although oxygen was toxic, our experiment unambiguously proved that *T. maritima* was able to consume it during a 12-hour exposure period. Furthermore, a shift in glucose metabolism towards lactate production was observed under oxidative conditions.

## 1. Introduction

Hyperthermophilic anaerobic bacteria similar to *Thermotoga* species should provide good models for generating new insights into the evolution and adaptation of anaerobes to oxidative conditions. Macromolecule-based [1, 2] and whole-genome-based analyses [3, 4] have indicated that *Thermotoga maritima* belongs to a slowly evolving lineage and is positioned deep within the bacterial domain of the phylogenic tree, making it a potential candidate as one of the ancient phenotypes [5]. *Thermotoga maritima* is a hyperthermophilic, fermentative, strict-anaerobic, and obligate chemo-organoheterotrophic organism able to use various sugars or complex organic substrates (e.g., on peptone) as carbon and energy sources [6–10]. It has been shown to ferment glucose into acetate, CO_2_, H_2_, and L(+) lactate, with the presence of L(+) lactate being dependent on culture conditions (e.g., H_2_ partial pressure) [6, 11, 12]. Similarly to most *Thermotoga *species, *T. maritima* reduces S° and thiosulfate to H_2_S [7, 11, 13]. Huber et al. [6] and later Schröder et al. [12] suggested that sulfur reduction in *Thermotoga* species should be considered a nonenergy-yielding electron-sink reaction preventing the accumulation of H_2_ as a means of “H_2_-detoxification.” 

*Thermotoga *spp. inhabit various hot ecosystems worldwide, including hot springs and deep-sea and shallow hydrothermal vents, and may therefore have to cope with the presence of oxygen in these ecological niches. Indeed, despite their strict anaerobic nature, geochemical and microbial analyses have demonstrated a significant abundance of *Thermotoga* species not only in anoxic but also partially-oxygenated hot sediments and fluids in hydrothermal vent ecosystems [14]. This suggests that these anaerobic bacteria must have developed biochemical mechanisms to deal with temporary exposures to oxygen. *T. neapolitana* has been shown to tolerate low-dissolved oxygen partial pressure [15, 16], and it was recently reported that *T. maritima* was able to grow in the presence of low oxygen concentrations, that is, 0.5% v/v in the gaseous phase of the culture [17]. Under these oxidative conditions, a flavoprotein homologous to the rubredoxin-oxygen oxidoreductase found in* Desulfovibrio s*pecies [18] was overproduced concomitantly with enzymes involved in reactive oxygen species detoxification, iron-sulfur-cluster synthesis/repair, and the cysteine biosynthesis pathway [17]. Furthermore, it has been reported that the gene encoding this rubredoxin-oxygen oxidoreductase belonged to a multicistronic unit including genes encoding proteins putatively involved in the biosynthesis of polysaccharides known as participating to biofilm formation [17]. These findings suggest that the defense strategy set up against oxygen in *T. maritima* includes two systems based on (i) oxygen-reducing enzymes eliminating intracellular oxygen and (ii) trapping the cells in the polysaccharide-matrix biofilm to minimize oxygen exposure [17]. Interestingly, exopolysaccharide production was observed in pure cultures of *T. maritima* [19, 20], but also in cocultures of *T. maritima* with *Methanococcus jannaschii* [19, 21]. These biofilm formations are known to be induced by a range of environmental factors, including pH, temperature, salt concentration, UV light, oxygen, and antibiotics [22, 23]. 

The aim of this study was to design a methodology for mimicking the physicochemical conditions and particularly the oxygen fluctuations likely to be encountered in hot marine environments marked by highly turbulent hydrodynamics. This methodology was used to study the effects of oxygen fluctuations on growth and glucose catabolism in* T. maritima.*

## 2. Materials and Methods

### 2.1. Strain, Media, and Culture Conditions

*Thermotoga maritima* strain MSB8 (DSMZ 3109) was used throughout the course of this work. All the media preparations described below were done using Hungate and Macy anaerobic techniques [24, 25]. *T. maritima* was cultivated on basal medium (BM) containing, per liter, NH_4_Cl 1 g, KH_2_PO_4_ 0.3 g, K_2_HPO_4_ 0.3 g, KCl 0.1 g, NaCl 25.0 g, MgCl_2_ 3.0 g, CaCl_2_ 0.1 g, Balch's trace mineral element solution [26] 10 mL, yeast extract 1.0 g. The medium was pH-adjusted to 7.0 with KOH 1 M and then boiled and cooled down to room temperature under a stream of O_2_-free N_2_. It was then distributed into 100-mL serum bottles (36 mL of medium) as previously described [27]. After sealing the serum bottles, the gaseous phase was flushed with a stream of O_2_-free N_2_ : CO_2_ (80 : 20% v/v). The medium was then autoclaved at 120°C for 20 min and stored at room temperature. Stock solutions of NaHCO_3_ (10% w/v), Na_2_S (2% w/v), cysteine-HCl (2.6% w/v) and glucose (1 M) were prepared under anoxic conditions as described by Miller [27] and stored under N_2_ : CO_2_ (80 : 20% v/v) atmosphere. Glucose solution was sterilized by filtration, while NaHCO_3_, cysteine-HCl, and Na_2_S solutions were sterilized by autoclaving (120°C for 20 min). Before inoculation, the medium was supplemented with 0.7 mL NaHCO_3_ (10% w/v) and 0.7 mL glucose (1 M) (final glucose concentration was 18.7 mM). To prepare BM medium under reducing conditions, the medium was supplemented with 0.7 mL Na_2_S (2% w/v) and 0.7 mL cysteine-HCl (2.6% w/v).

All serum-bottle cultures of *T. maritima* were incubated in an incubator shaker (Infors HT Thermotron, Switzerland) at 80°C and 130 rpm. Cultures were performed in triplicate for each experiment.

### 2.2. Experimental System

*Thermotoga maritima* was batch-cultured in a 2.3-L double-jacket glass bioreactor (FairMenTec, France) with a 1.5-L working volume. The fermentor was run with stirring driven by two axial impellers, and was equipped with sensors monitoring temperature (Prosensor pt 100, France), pH (Mettler Toledo InPro 3253, Switzerland), redox potential (Mettler Toledo InPro 3253, Switzerland) and pO_2_ (Mettler Toledo InPro 6800, Switzerland). The inlet gaseous stream consisting of a mixture of O_2_ and N_2_ was prepared using two mass-flow meters (Aalborg GFC17, range 0–100 and 0–10 SCCM, USA). This stream was injected through a nozzle immersed in the bioreactor. The steam in the outlet gas stream was condensed into a glass exhaust water cooler (temperature-controlled at 4°C with a cooling bath equipped with a pump (Julabo SE 6, France) to prevent liquid loss in the bioreactor (water vapor condensates were returned to the culture vessel). On the outlet gas stream line, a CARBOCAP carbon dioxide probe (Vaisala GMP221, Finland) connected to a transmitter (Vaisala Series GMT221, Finland) was fitted downstream of the glass exhaust water cooler. This system allowed online measurement of CO_2_ content within the outlet gas stream. To prevent air from entering the bioreactor, the outlet gas stream line was closed at the end by a hydraulic seal (2 cm-deep immersion in an oil reservoir). The bioreactor was heated via hot-oil circulation into the double jacket using a heat bath equipped with a pump (Julabo F25, France). Bioreactor liquid volume and NaOH consumption, which was used to regulate culture pH, were tracked by two balances (Sartorius Combics 1 and BP 4100 (France), resp.). Temperature, pH, gaseous-stream flow rates and stirrer speed were regulated through control units (local loops). All this equipment was connected to a Wago PLC (France) via a serial link (RS232/RS485), a 4–20 mA analog loop, or a digital signal. The PLC was connected to a computer for process monitoring and data acquisition. BatchPro software (Decobecq Automatismes, France) was used to monitor and manage the process with good flexibility and total traceability.

### 2.3. Operating Conditions

All the bioreactor experiments were performed using the anaerobic BM containing glucose (20 mM). Step 1: the bioreactor was filled with 1350 mL of MS (pH about 6.2) and then sterilized by autoclaving at 120°C for 20 min. Before each fermentation run, pH and redox probes were separately calibrated at 80°C with pH 4.22 and 7.04 buffers (Mettler Toledo, Switzerland) and a 124 mV redox buffer at pH 7.0 (Mettler Toledo, Switzerland), respectively. The pO_2_ probe was accurately calibrated using air sparging into MS maintained at 80°C. Twenty-one percent oxygen (air composition) represented 100% of the saturation scale and corresponded to 90 *μ*M O_2_ in water in presence of 25 g L^−1^ NaCl. Step 2: anoxia was established by flushing the bioreactor overnight with an O_2_-free N_2_ stream at 20 mL min^−1^. The medium was then supplemented with anoxic sterile solutions of glucose (3 M), MgCL_2_ (900 g L^−1^) and yeast extract (300 g L^−1^) to obtain final concentrations as reported for BM. Step 3: the bioreactor was inoculated with 160 mL of overnight culture, performed with anoxic BM, and flushed for 30 min with an O_2_-free N_2_ stream at 100 mL min^−1^. Before starting fermentation runs, total culture volume was 1530 mL. During the fermentation run, the O_2_-free N_2_ stream used as carrier gas was cut to 3 mL min^−1^. For all fermentation runs, temperature and stirrer speed were regulated at 80°C ± 0.5°C and 150 ± 5 rpm, respectively. pH was regulated at 7.0 ± 0.1 by addition of NaOH 1 M. At the end of each fermentation run, the fermentation medium was acidified to pH 1.5–2.0 with concentrated H_2_SO_4_ to determine the CO_2_ volume represented by HCO_3_^−^ and CO_3_^2−^ ions.

The oxidative period performed during the stationary growth phase included four short oxygen pulses followed by one long oxygen pulse then a further four short oxygen pulses in order to simulate the oxidative stress conditions potentially encountered by *Thermotoga maritima* in its ecological niches. The first four oxygen pulses consisted in a total flow rate (100 mL min^−1^) of N_2_ : O_2_ gaseous mixture: (i) O_2_ : N_2_ 4 : 96 (% v/v) for 24.4 min (dissolved oxygen (DO) reached 13.4 *μ*M), (ii) O_2_ : N_2_ 2 : 98 (% v/v) for 6 min (DO reached 6.3 *μ*M), (iii) O_2_ : N_2_ 9 : 91 (% v/v) for 3.7 min (DO reached 36.1 *μ*M), and (iv) O_2_ : N_2_ 1.5 : 98.5 (% v/v) for 2 min (DO reached 2.9 *μ*M). The second long oxygen pulse was performed with a total flow rate of 4 mL min^−1^: O_2_ : N_2_ 5 : 95 (% v/v) for 14 hours (DO reached 2.4 *μ*M at completion). The last four short pulses consisted in a total flow rate of 100 mL min^−1^: (i) O_2_ : N_2_ 5 : 95 (% v/v) for 4.4 min (DO reached 16.0 *μ*M), (ii) O_2_ : N_2_ 8 : 92 (% v/v) for 5.6 min (DO reached 68.7 *μ*M), (iii) O_2_ : N_2_ 2 : 98 (% v/v) for 3.2 min (DO reached 8.5 *μ*M), and (iv) O_2_ : N_2_ 1 : 99 (% v/v) for 6.1 min (DO reached 4.2 *μ*M). For all short oxygen pulses, after each oxygen injection, when the set point for dissolved O_2_ concentration was reached, O_2_ flow and agitation were stopped until DO was close to zero micromoles. Furthermore, before each oxygen injection, the bioreactor headspace was flushed with N_2_ at a flow rate of 100 mL min^−1^ for 5 minutes to restore anoxic conditions. Moreover, for the long oxygen pulse, stirring was held at a constant 150 rpm for the total oxidative period.

### 2.4. Analytical Methods

Cultures performed under anoxic conditions revealed trace amounts of mineral matter in suspension. Thus, all culture samples (900 *μ*L) were supplemented with 100 *μ*L of a 150 mM acetic acid (pH 4) solution to solubilize the mineral suspensions before measuring optical density (OD). OD was determined in triplicate at 600 nm using an S2100 Diode array UV-visible spectrophotometer (WPA Biowave, France). One unit of OD at 600 nm corresponded to 0.339 g cell dry weight L^−1^. Bacterial growth in the bioreactor was also determined by direct cell counting using a Thoma chamber (depth: 0.02 mm) and a 100 × phase contrast microscope (Leica DME, France). Countings were performed in triplicate.

During fermentation, nitrogen, oxygen and hydrogen contents were measured at regular intervals by withdrawing 1-mL samples from the bioreactor headspace into gas-tight syringes and injecting these subsamples into a TCD-GC system (Shimadzu 8A, Japan) equipped with a concentric CTR1 column (Alltech, USA). This system was connected to a computer running WINILAB III software (Perichrom, France). Operating conditions were as follows: oven temperature 35°C; detector and injector temperature 100°C; current 60 mA; argon carrier gas at 60 mL min^−1^. During fermentation, glucose, acetate, lactate and fructose concentrations were determined by HPLC. One mL of culture sample was centrifuged for 5 minutes at 14,500 rpm, then 20 *μ*L was loaded onto an Animex HPX-87H column (Biorad) set at 35°C and eluted at 0.5 mL min^−1^ with H_2_SO_4_ solution (0.75 mM). Product concentrations were determined with a differential refractometer detector (Shimadzu RID 6 A, Japan) connected to a computer running WINILAB III software (Perichrom, France). All analyses were repeated in triplicate. Accuracy of the measured quantities of glucose, acetate, lactate and fructose was 2%. L-alanine and *α*-aminobutyrate concentrations were determined by HPLC as described by Moore et al. [28].

Microbial extracellular polysaccharides (EPS) were quantified by colorimetry [29]. Throughout this paper, EPS values are converted to glucose equivalent, expressed in millimoles. The accuracy of the measured quantities of EPS achieved in this study was about ±0.7 mM of glucose equivalent.

### 2.5. Determination of H_2_ and CO_2_ Production Rates

The detection of the gaseous compounds (CO_2_ and H_2_) produced in the bioreactor during the culture of *T. maritima *requires a low N_2_ (carrier gas) flow rate (3 mL min^−1^). During the experiments, N_2_ flow rate was kept constant and is thus considered reference gas for calculating the partial pressures of CO_2_ and H_2_ in the bioreactor outlet gas stream. To determine the production of H_2_ and CO_2_, we developed a mathematical model based on the material balances of the 3 gaseous compounds (N_2_, H_2_ and CO_2_) as follows:

(1) $$\begin{array}{c} \frac{dp_{{\text{N}}_{2}}^{\text{out}}}{dt}=\frac{Q_{{\text{N}}_{2}}}{\left(V_{\text{HR}}-V_{\text{Steam}}\right)}\times p_{{\text{N}}_{2}}-\frac{Q_{T}^{\text{out}}}{\left(V_{\text{HR}}-V_{\text{Steam}}\right)}\times p_{{\text{N}}_{2}}^{\text{out}},\\ \frac{dp_{{\text{CO}}_{2}}^{\text{out}}}{dt}=\frac{Q_{{\text{CO}}_{2}}}{\left(V_{\text{HR}}-V_{\text{Steam}}\right)}\times p_{{\text{CO}}_{2}}-\frac{Q_{T}^{\text{out}}}{\left(V_{\text{HR}}-V_{\text{Steam}}\right)}\times p_{{\text{CO}}_{2}}^{\text{out}},\\ \frac{dp_{{\text{H}}_{2}}^{\text{out}}}{dt}=\frac{Q_{{\text{H}}_{2}}}{\left(V_{\text{HR}}-V_{\text{Steam}}\right)}\times p_{{\text{H}}_{2}}-\frac{Q_{T}^{\text{out}}}{\left(V_{\text{HR}}-V_{\text{Steam}}\right)}\times p_{{\text{H}}_{2}}^{\text{out}},\\ Q_{T}^{\text{out}}\;=\;Q_{{\text{N}}_{2}}\;+Q_{{\text{CO}}_{2}}+\;Q_{{\text{H}}_{2}},\\ p_{{\text{N}}_{2}}^{\text{out}}+p_{{\text{CO}}_{2}}^{\text{out}}+p_{{\text{H}}_{2}}^{\text{out}}=100\text{\%}\;\text{or}\;1\;\text{bar}\;\left(\text{atmospheric}\;\text{pressure}\right) \end{array}$$

where *p*_N_2__^out^, *p*_CO_2__^out^, and *p*_H_2__^out^ are the partial pressures of N_2_, CO_2_, and H_2_, respectively, in the outlet gas stream, *p*_N_2__= *p*_CO_2__= *p*_H_2__= 100% are the partial pressures of N_2_ (carrier gas), CO_2_, and H_2_ (biological gas produced during fermentation), respectively, *V*_HR_ (543 mL) is bioreactor headspace volume, and *V*_Steam_(161 mL) is water vapor volume. Vapor volume was calculated according to the Antoine equation at 68.8°C (median headspace temperature during the fermentation run). *Q*_N_2__, *Q*_CO_2__, *Q*_H_2__, and *Q*_*T*_^out^ are the N_2_, CO_2_, and H_2_ flows and the sum of these three gases, respectively. At 68.8°C (in headspace temperature), carrier gas flow rate *Q*_N_2__ was 3.5 mL min^−1^ and was calculated as follows: 3.0 mL min^−1^ × (273 + 68.8°C)/(273 + 20°C).

### 2.6. Fermentation Parameters

Cellular yield on glucose (Y_x/glu_) was determined using only the proportion of glucose metabolized.

## 3. Results

### 3.1. Design of an Anoxic Medium without Reducing Agent for Cultivating *T. maritima*

Growth kinetics experiments performed in serum bottles showed that in the BM containing 1.0 or 0.5 g L^−1^ of yeast extract, maximum cellular concentrations were obtained in the presence of reducing agents (0.4 and 0.5 g L^−1^ of Na_2_S and cysteine, resp.). Indeed, maximum optical densities (OD) were obtained in presence of reducing agents (0.6 *versus* 0.3 and 0.3 *versus* 0.15 in the presence of 1.0 and 0.5 g L^−1^ of yeast extract, resp.). This result highlighted that the redox potential of the culture medium was critical for *T. maritima* growth and that yeast extract at concentrations below 1.0 g L^−1^ limited growth. In order to study the effect of the presence of oxygen on *T. maritima* growth, all the following bioreactor experiments were performed using BM formulated with 1 g L^−1^ yeast extract without reducing agent supplementation so as to limit the chemical reduction of oxygen.

### 3.2. Growth and Metabolism of *T. maritima* under Anoxic Conditions

**Figure 1:**
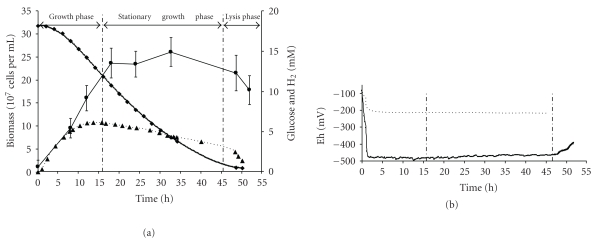
Growth of *Thermotoga maritima* cultivated in a bioreactor under anoxic conditions: (a) biomass (*⬤*), glucose (*◆*), H_2_ production (▲) and (b) redox potential (Eh) of noninoculated medium (discontinuous line) and *T. maritima*-inoculated medium (continuous line).

All experiments were now performed in bioreactors. The growth pattern illustrated in Figure 1 consisted of three phases: (i) growth phase for 16 hours, (ii) stationary phase from 16 to 47 hours, and (iii) lysis phase from 47 to 52 hours. Over the first two hours of the growth phase, the redox potential (Eh) of the fermentation medium dropped sharply from about −85 mV to −480 mV, and thereafter remained low and roughly constant (−480 mV to −450 mV) until the end of the stationary phase (Figure 1(b)). However, during the lysis phase, Eh increased sharply from −460 to −400 mV concomitantly with glucose exhaustion and/or cell lysis (Figure 1). In control experiments (noninoculated culture medium), Eh dropped to only −200 mV, probably due to the presence of reducing compounds (e.g., cysteine) in the yeast extract (Figure 1(b)). The difference between controls and culture led us to conclude that the significant reductive process was due to *T. maritima* cell activity (Figure 1). 

During the total fermentation period, fermentation balance indicated that glucose was fermented (O/R close to 1.0), and 87.8 ± 10% of glucose consumed was converted into acetate, lactate, CO_2_, H_2_, L-alanine, fructose, and exopolysaccharides (EPSs) (Table 1). Analyses to detect other metabolites revealed traces of *α*-aminobutyrate. In addition, only trace amounts of acetate (<1 mM) were produced by *T. maritima* from yeast extract utilization when grown on BM in the absence of glucose. Among the end-products resulting from glucose fermentation, acetate, lactate, CO_2_, and L-alanine accounted for 70.5 ± 6% of all carbon-glucose consumed, while EPS and fructose represented 8.0 ± 4% and 9.3 ± 0.4%, respectively (Table 1). The presence of fructose, which *T. maritima* can also use as energy source, probably ensued from chemical isomerization of glucose at high temperatures, as observed in abiotic controls (data not shown). During this control experiment, it was shown that glucose was not chemically destroyed at 80°C over 50 hours under anoxic conditions. 

In addition, the close match between, on one hand, the calculated molar ratios of H_2_/CO_2_, H_2_/acetate, and CO_2_/acetate (1.94, 2.11 and 1.09, resp.) and, on the other hand, the theoretical stoichiometry of the anaerobic acetate production pathway (2.0, 2.0 and 1.0, resp.) clearly demonstrated that the H_2_ and CO_2_ flow rates produced during glucose fermentation were correctly estimated, thus validating the methodology used (see Section 2.5).

Cross-comparison of the fermentation parameters for the growth phase and the stationary growth phase revealed that volumic glucose consumption (Q_glu_) remained roughly unchanged (0.39 and 0.36 mM h^−1^ for the growth and stationary growth phases, resp.; Table 3) whereas specific glucose uptake (q_glu_) was higher in the growth phase than the stationary growth phase (9.3 and 3.3 mmoles glucose g cdw^−1^ h^−1^, resp.). Furthermore, the proportion of glucose fermented into acetate, lactate, CO_2_, H_2_, and L-alanine was almost constant and fairly independent of growth status, as established by “acetate + lactate + L-alanine + CO_2_/glucose consumed” ratios (73.8 and 68.6% for growth and stationary growth phases, resp.) (Table 3). On the other hand, although the standard deviation for the EPS measurements was particularly high, EPSs were mainly produced during the growth phase (1.39 mM), representing about 22.3 ± 11% of carbon-glucose consumed (Table 1).

### 3.3. Effect of Oxidative Conditions on *T. maritima* Growth and Metabolism

To study the effect of oxygen on *T. maritima* metabolism, oxygen fluxes were introduced into the bioreactor after the growth phase performed under anoxic conditions. The oxygen addition protocol consisted of a first sequence of (i) four short pulses of oxygen for 66 minutes, (ii) a second long pulse of oxygen for 14 hours, and (iii) a third sequence of four short pulses of oxygen for 54 minutes (see Section 2 for details). A prior experiment performed with a noninoculated culture medium (control experiment) under the same oxygen input flow in the bioreactor indicated that 0.64 mM of glucose (from 20 mM initially present) was chemically destroyed over 30 hours at 80°C.

**Figure 2:**
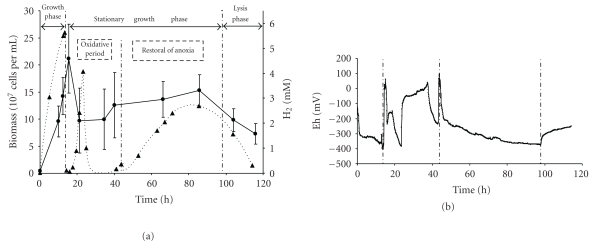
Growth of *Thermotoga maritima* cultivated in a bioreactor under anoxic conditions including oxidative period: (a) biomass (*⬤*) and H_2_ production (▲), and (b) redox potential (Eh).

**Figure 3:**
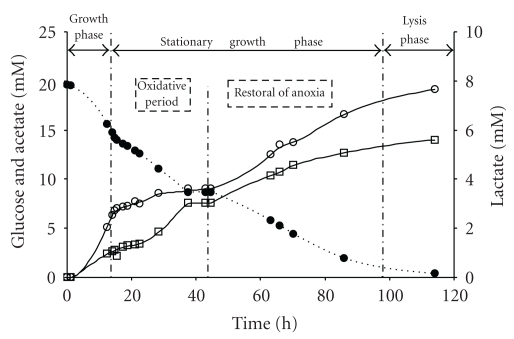
Fermentative parameters of *Thermotoga maritima* cultivated in a bioreactor under anoxic conditions including oxidative period: glucose (*⬤*), acetate (*⚪*), and lactate (□).

In order to clearly present our analysis of fermentation results, the patterns of different fermentation parameters illustrated in Figures 2 and 3 were divided into four phases, corresponding to (i) the growth phase (14 hours), (ii) the stationary growth phase including one global oxidative period (14 to 44.6 hours), followed by (iii) a restoral of initial anoxic conditions (44.6 to 97 hours), and (iv) the lysis phase from 97 to 114.2 hours.

**Figure 4:**
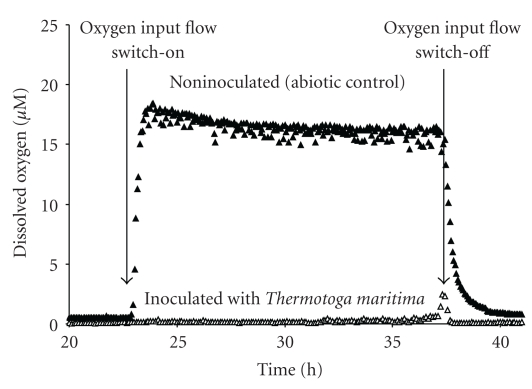
Dissolved oxygen measurements (▲) in a noninoculated bioreactor used as a control and (Δ) in a bioreactor inoculated with *T. maritima*, during the long oxygen pulse of the oxidative period (between 23 and 37 hours). The arrows indicate the oxygen input flow switch-on and switch-off, respectively.

The fermentation results obtained for the growth phase performed under anoxic conditions (Tables 2 and 3) were similar to the results analyzed above. In order to demonstrate oxygen consumption by *T. maritima*, we elected to show the dissolved oxygen (DO) data during the long pulse of oxygen (between 23 and 37 hours of fermentation run) (Figure 4) corresponding to an input gas flow of O_2_ 5 % (v/v) in the bioreactor. These measurements were compared with those obtained under the same conditions in a noninoculated medium used as control (Figure 4). In abiotic controls, measured DO in BM in the absence of yeast extract was 22.5 *μ*M. This value decreased to 16 *μ*M in the same culture medium in the presence of yeast extract (YE), thus demonstrating that O_2_ was poorly chemically reduced due to YE supplementation (Figure 4). However, in the presence of *T. maritima*, O_2_ concentrations remained close to zero *μ*M over 12 hours during the long pulse of oxygen (Figure 4). This result unambiguously indicated that O_2_ was totally consumed by *T. maritima* culture during this long oxygen pulse period.

Furthermore, after the first series of four short oxygen pulses, cell counts and OD levels fell dramatically as a result of biomass flocculation (Figure 2(a)). Thus, the heterogeneousness of the culture made any measurement of cell counts, OD or polysaccharide concentrations (data not shown) chaotic and unreliable. However, while *T. maritima* was able to consume the oxygen, its presence was nevertheless toxic, as established by the significant reducing of glucose consumption rate (Q_glu_ was 0.36 and 0.16 mM h^−1^ under anoxic and oxidative conditions, resp.; Table 3). Furthermore, despite restoral of anaerobic conditions after the oxidative period, Q_glu_ still remained low (0.12 mM h^−1^; Table 3). Consequently, total duration of fermentation increased from 47 hours for the anoxic fermentation run to 114 hours for the fermentation run including the oxidative period (Figures 1 and 3). Exposing *T. maritima* to oxygen during the stationary growth phase clearly resulted in a decrease in (i) the proportion of glucose fermented into acetate, lactate, CO_2_, and L-alanine, and (ii) a significant shift in glucose catabolism towards lactate, as established by the ratios (i) (acet + lac + ala + CO_2_)/glu (68.6 ± 4.5 *versus* 56.1 ± 3.5% of carbon-glucose consumed), and (ii) (lac/acet) (0.26 ± 0.01 *versus* 0.78 ± 0.03), respectively (Table 3). It should be noted that after the oxidative period and total restoral of anoxic conditions, although glucose consumption rate (Q_glu_) decreased (Table 3), *T. maritima* recovered its fermentative pattern. Indeed, the (acet + lac + ala + CO_2_)/glu and (lac/acet) ratios calculated for the anoxic stationary growth phase concerning (i) the anoxic fermentation run (68.6 ± 4.5 and 0.26 ± 0.01, resp.; Table 3) and (ii) the anoxic fermentation run including the oxidative period (76.6 ± 5.2 and 0.25 ± 0.01, resp.; Table 3) were similar.

## 4. Discussion

### 4.1. Capacity of Thermotoga Maritima to Reduce the Culture Medium

Using bioreactor experiments performed with anoxic BM in the absence of reducing agents, we demonstrated that *T. maritima* growing with glucose as energy source was able to reduce the culture medium by itself, as shown by the sharp decrease in redox potential (Eh) down to a range of −450 to −480 mV. The lowest redox potentials in nature have been measured in anoxic environments dominated by strict anaerobes such as methanoarchaea, clostridia and sulfate- or sulfur-reducing bacteria. Looking at methanoarchaea, it is a paradigm that methane production is only possible under anoxic environmental conditions with an Eh of less than −200 to −400 mV [30–32]. In *Methanosarcina barkeri*, Jee et al. [33] studying Eh-stat batch cultures using methanol as carbon and energy source showed that methane production rates were optimal under a redox potential range of −430 to −520 mV. Moreover, Fetzer and Conrad [34] reported that when cultivated on methanol, this same archaeon also reduced the culture medium by itself, mirroring the pattern here for *T. maritima*. The mechanism by which *T. maritima* was able to reduce the medium remains unclear. A barrier to understanding this phenomenon is to identify the redox couples involved, such as the organic or mineral compound(s) used as electron acceptors or the nature of the biological electron donors. On this point, it should be noted that *T. maritima* was able to grow on hydrogen in presence of Fe^3+^ as final electron acceptor [35], and various flavoproteins and iron-sulfur proteins have been identified as potential electron carriers [4]. In addition, our results further suggested that the ability of *T. maritima* to reduce the medium was dependent on its catabolic activity. Indeed, redox potential decreased and remained low as long as glucose was available, whereas it increased sharply after glucose exhaustion and cell lysis.

### 4.2. Growth and Glucose Catabolism in *T. maritima* Cultivated in Anoxic BM Medium

In bioreactor cultures of *T. maritima* within the anoxic BM in the absence of reducing agents, growth yield was estimated at about 16.7 ± 4 g cdw mole^−1^ of glucose. This estimation was significantly lower than that reported by Schröder et al. [12] who estimated the growth yield in *T. maritima* at 45 g mole^−1^ when it was cultivated with a medium containing Na_2_S and cysteine-HCl as reducing agents together with 0.5 g L^−1^ of yeast extract. These findings therefore confirmed that *T. maritima* prefers to grow not only in strictly anoxic but also in reduced conditions, as previously reported [6]. Taking into account these results, it could be hypothesized that when *T. maritima* was cultivated in BM in the absence of reducing agents, a significant portion of the energy produced by cells could be used to decrease the Eh of the culture medium, and keep it at low levels. 

Focusing on glucose catabolism in *T. maritima*, bioreactor experiments performed under anoxic conditions showed that glucose was converted mainly into acetate, lactate, H_2_ and CO_2_ (65.9 ± 6% of carbon-glucose consumed), but also to a lesser extent into exopolysaccharide (EPS), L-alanine (8.0 ± 4% and 4.6% of carbon-glucose consumed, resp.) and trace amounts of *α*-aminobutyrate, as already reported for *T. lettingae* [36]. These results are consistent with previous reports on *T. maritima *studying L-alanine production from glucose metabolism [37]. Furthermore, cross-comparison of fermentation product balances analyzed specifically in the growth and stationary growth phases suggested that EPS were mainly produced during the growth phase, representing about 22.3 ± 11% of carbon-glucose consumed. This same pattern of *T. maritima* production was previously reported by Rinker and Kelly (2000) in chemostat experiments on *T. maritima* with maltose as energy source, where EPS yield varied from 4% to 10% depending on growth rate [20]. In our case, the higher EPS yield rate could be due to an excess glucose concentration for almost the full duration of the in-batch fermentation run, whereas in the chemostat cultures used by Rinker and Kelly [20], growth was limited by the energy source.

### 4.3. Effect of Oxygen on Glucose Catabolism in *T. maritima* during the Stationary Growth Phase

Although *T. maritima* was originally described as strictly anaerobic, this bacterium and other *Thermotoga *spp. are significantly abundant in all marine hydrothermal fluids and sediments, even when partially oxygenated from subaerial and shallow submarine sites such as Vulcano Island [14]. We therefore conducted studies using a novel experiment unit and adapted the methods to mimic the oxygen fluctuations most likely to occur in the original ecosystem from where *T. maritima* originated (e.g., deep-sea hydrothermal vents exposed to spatial and temporal variations in oxygen) [38]. 

In terms of the toxic effect of oxygen on *T. maritima*, we clearly demonstrated that the presence of oxygen drastically reduced glucose consumption rate (see Q_glu_ in Table 3), leading to a significant increase in fermentation time (Figures 2 and 3). Furthermore, comparison of the glucose consumption rates (Q_glu_ in Table 3) estimated for the stationary phase in both fermentation runs (with and without an oxidative period) showed that after oxygen exposure and total restoral of anoxic conditions, Q_glu_ fell from 0.36 down to 0.12 mM h^−1^. In this respect, based on both these Q_glu_ values (0.36 and 0.12 mM h^−1^), we can hypothesize that about two thirds of the *T. maritima* cells had probably been inactivated and/or lysed after the oxidative period. However, although the presence of oxygen was toxic for *T. maritima* growth, our results unambiguously demonstrated that *T. maritima* was able to totally consume oxygen over a 12-hour exposure (long oxygen pulse), as confirmed by results obtained from experiments conducted in the presence or absence of this bacterium (Figure 4). Our results on oxygen consumption by *T. maritima* are in agreement with genome analysis of this bacterium. Indeed, several genes have been detected that encode two NADH oxidases and a putative alkyl hydroperoxide reductase [4] known to be oxygen-detoxifying enzymes. Focusing on these enzymes, Yang and Ma [39] demonstrated that a NADH oxidase purified from *T. maritima* catalyzed the reduction of oxygen to hydrogen peroxide. This enzyme activity could therefore be coupled to the activity of hydrogen peroxide-scavenging enzymes, consequently removing the oxygen. More recently, Le Fourn et al. [17] reported that *T. maritima* cultivated in presence of oxygen overproduced (i) enzymes involved in ROS detoxification, iron-sulfur center synthesis/repair, and the cysteine biosynthesis pathway and (ii) a flavoprotein homologous to rubredoxin : oxygen oxidoreductase from *Desulfovibrio* species. These authors demonstrated that the flavoprotein exhibited an oxygen reductase activity that could also account for oxygen consumption in *T. maritima* [17].

Concerning the effect of oxygen on the fermentative pattern of *T. maritima *in the stationary growth phase, the presence of oxygen decreased the proportion of glucose fermented into acetate, lactate, CO_2_, H_2_, and L-alanine from 68.6 ± 4.5 to 56.1 ± 3.5% and significantly shifted glucose fermentation towards lactate production. This shift in catabolism could possibly be explained by a very oxygen-sensitive hydrogenase enzyme. Indeed, hydrogen production in *T. maritima* can be regarded as a metabolic pathway for releasing excess reducing equivalents, thereby maintaining intracellular redox equilibrium. In this respect, the reduction of pyruvate to lactate could be seen as an alternative pathway that is stimulated in presence of oxygen. Furthermore, analyses to detect other metabolites during this oxidative period revealed that there was no significant modification in the production of L-alanine or *α*-aminobutyrate from glucose metabolism compared to *T. maritima* growth under anaerobic conditions. Flocculation problems encountered in our experimental operating conditions when oxygen was introduced into the bioreactor unfortunately made it impossible to accurately quantify the amount of EPS produced by *T. maritima*. However, this inaccuracy in quantifying EPS can be extended to experiments conducted under anoxic conditions. Therefore, further specific experiments are needed to address the issue of EPS detection in *T. maritima* cultures performed in our experimental conditions (e.g., extraction, purification, and detection of polysaccharides from larger culture volumes). In this respect, this study highlighted that, under oxidative growth conditions, the carbon recovery balance of* T. maritima *fermentation was highly dependent on EPS measurements. This point is emphasized, since EPS have been suggested to play a significant role in the defense strategy employed by *T. maritima *to cope with oxygen toxicity or other environmental constraints [17, 20, 22, 40]. Interestingly, Le Fourn et al. (2008) reported biofilm formation in *T. maritima* when cultivated in presence of oxygen. They observed that the oxygen reductase-encoding gene induced by the presence of oxygen belonged to a multicistronic unit that included genes encoding proteins involved in EPS biosynthesis [17].

Finally, our results indicated that the broad ubiquity of *T. maritima*, and most probably other members of the order *Thermotogales *in contact with oxygen in hot environments may be explained by their ability to create their own hospitable redox environment, to consume oxygen to a certain extent, and ultimately to cope with limited amounts of oxygen. Interestingly, these physiological features are also partially shared by methanoarchaea [34, 41] and sulfate-reducing bacteria, which are known as ubiquitous strict anaerobes [42]. This work also allowed us to validate a novel experimental culture unit with instruments adapted to studying the physiology of not only anaerobic but also aerotolerant or microaerophilic hyperthermophiles, at 80°C. The validation of oxygen measurements in the culture medium at both low oxygen concentration and high temperature will be helpful in improving our knowledge of how such microorganisms cope with the fluctuating oxidative conditions they have to face in the hot environments they inhabit.

## Figures and Tables

**Table 1:** Batch balance for *Thermotoga maritima* grown on BM under anoxic conditions.

Phase	Glucose consumed (mM)	Biomass (mg cdw L^−1^)	Fructose produced (mM)	End-products of metabolism (mM)						Carbon balance (%)
				Acetate	Lactate	Alanine	EPS	CO_2_	H_2_	
Growth	6.24	83.4 ± 17	1.24	6.51	1.34	0.9 ± 0.2	1.39 ± 0.7	7.90	13.24	116.0 ± 18
Stationary growth	11.17	24.3 ± 17	0.38	11.54	3.02	0.7 ± 0.2	0.0 ± 0.7	11.74	24.85	72.0 ± 11
Overall fermentation	17.41	107.7 ± 17	1.62	18.05	4.36	1.6 ± 0.2	1.39 ± 0.7	19.64	38.09	87.8 ± 10

Fermentation runs were conducted at 80°C under anoxic conditions using the BM.

Growth phase (0–15.9 h); stationary growth phase (15.9–47 h).

cdw: cell dry weight.

EPS: Extracellular polysaccharides.

**Table 2:** Batch balance for *Thermotoga maritima* grown on BM under anoxic conditions including the oxidative period.

Phase	Oxygen addition	Glucose consumed (mM)	Biomass (mg cdw L^−1^)	Fructose produced (mM)	End products of metabolism (mM)						Carbon balance (%)
					Acetate	Lactate	Alanine	EPS	CO_2_	H_2_	
Growth	−	5.46	81.4 ± 17	1.10	5.60	0.91	0.7 ± 0.2	1.22 ± 0.7	6.53	9.91	111.4 ± 19
Oxidative stationary growth	+	4.86	−	0.71	2.52	1.96	0.1 ± 0.2	nd	5.13	3.22	70.7 ± 18
Stationary growth	−	8.30	−	0.00	10.15	2.58	0.5 ± 0.2	nd	8.63	18.62	76.3 ± 13
Overall fermentation		19.3	81.4 ± 17	1.81	18.27	5.45	1.3 ± 0.2	1.22 ± 0.7	20.29	31.75	88.7 ± 11

Fermentation runs were conducted at 80°C under anoxic conditions using the BM.

Growth phase (0–14 h); oxidative stationary growth phase (14–44.6 h); anoxic stationary growth phase (44.6–114.2).

cdw: cell dry weight.

EPS: Extracellular polysaccharides.

**Table 3:** Comparison of fermentative parameters of *Thermotoga maritima* during the anoxic fermentation run and the anoxic fermentation run including oxidative period.

Fermentation conditions	Anoxic run		Anoxic run including oxidative period		
Phase	Growth	Stationary	Growth	Stationary	
Conditions	Anoxic	Anoxic	Anoxic	Oxidative	Anoxic
Q_glu_ (mM h^−1^)	0.39 ± 0.04	0.36 ± 0.01	0.39 ± 0.04	0.16 ± 0.02	0.12 ± 0.003
(acet + lac + ala + CO_2_)/(glu consumed) (%C)	73.8 ± 4.5	68.6 ± 4.5	68.9 ± 5	56.1 ± 3.5	76.6 ± 5.2
lac/acet (mole mole^−1^)	0.20 ± 0.01	0.26 ± 0.01	0.16 ± 0.01	0.78 ± 0.03	0.25 ± 0.01

Q_glu_: glucose consumption rates.

(acet + lac + ala + CO_2_)/(glu consumed): ratio of acetate + lactate + L-alanine + CO_2_ produced on glucose consumed.

lac/acet: ratio of lactate produced on acetate produced.
